# Anion Ordering and Phase Stability Govern Optical Band Gaps in BaZrS_3*x*_Se_3–3*x*_

**DOI:** 10.1021/acs.chemmater.6c01949

**Published:** 2026-09-22

**Authors:** Erik Fransson, Michael Xu, Prakriti Kayastha, Kevin Ye, Ida Sadeghi, Rafael Jaramillo, James M. LeBeau, Lucy Whalley, Paul Erhart

**Affiliations:** † Department of Physics and Astronomy, 11248 Chalmers University of Technology, SE-41296 Gothenburg, Sweden; ‡ Department of Materials Science and Engineering, 2167 Massachusetts Institute of Technology, Cambridge, Massachusetts 02139, United States; § School of Engineering, Physics and Mathematics, 5995 Northumbria University, Newcastle upon Tyne NE1 8QH, United Kingdom; ∥ Department of Chemistry, 4919 University College London, London WC1E 6BT, United Kingdom

## Abstract

Chalcogenide perovskites have emerged as promising lead-free materials for photovoltaic and thermoelectric applications. Among them, BaZrS_3_ has attracted particular attention due to its thermal and chemical stability, favorable optoelectronic properties, and low thermal conductivity. Here, we combine molecular dynamics and Monte Carlo simulations based on a machine-learned interatomic potential with scanning transmission electron microscopy to investigate mixing thermodynamics and phase stability in the BaZrS_3*x*_Se_3–3*x*_ system. We identify an unusual ordered structure, with signatures of anion ordering persisting at room temperature, most prominently at 33% S, where S and Se atoms form alternating layers within the crystal. Free-energy calculations yield the temperature–composition phase diagram, including a nonperovskite δ phase in the Se-rich limit and a perovskite phase in the S-rich limit, separated by a broad two-phase region. Analysis of the dielectric function and the absorption coefficient demonstrates that composition, crystal structure, and anion ordering jointly control the optical band gap. Selenium alloying enables tuning between approximately 1.5 and 1.9 eV, while anion ordering within a given composition reduces the gap by about 0.2 eV. Lastly, variations between structural polymorphs give rise to band gap differences of up to 0.5 eV.

## Graphical abstract

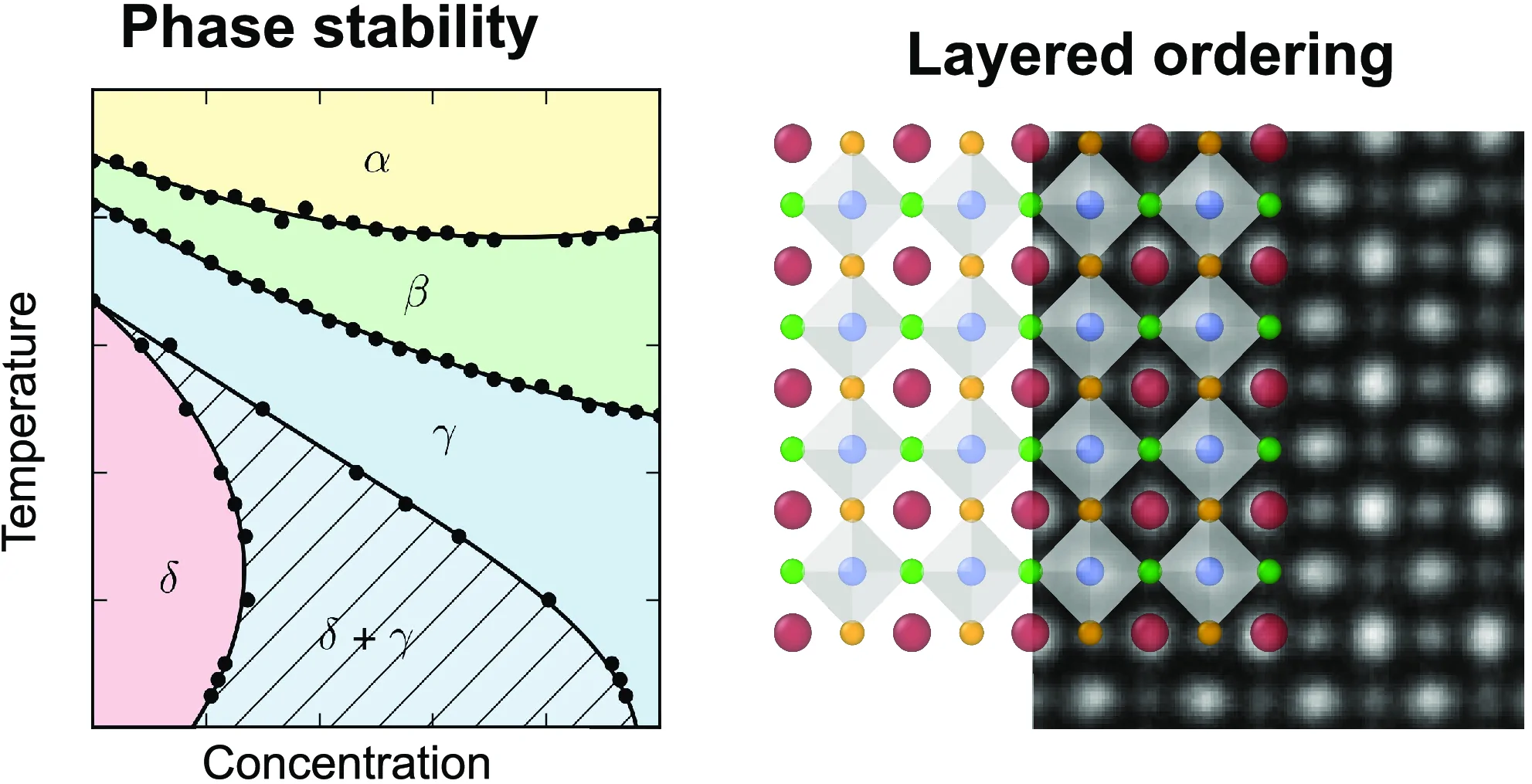

## Introduction

Perovskites with the general formula ABX_3_ constitute a versatile class of materials for optoelectronic applications. Hybrid lead–halide perovskites, in particular, have enabled record-breaking single-junction and tandem photovoltaic efficiencies.^1^ However, their long-term chemical instability and the presence of toxic lead remain significant barriers to large-scale deployment. These limitations have motivated the search for alternative perovskite chemistries that retain favorable optoelectronic properties while improving chemical robustness and sustainability.

Chalcogenide perovskites (X = S, Se) provide one such platform. These materials combine strong optical absorption with improved environmental stability and are composed of earth-abundant, nontoxic elements.^2–7^ Among them, BaZrS_3_ has emerged as a model system, exhibiting strong band-to-band photoluminescence and favorable defect physics.^8–11^ However, its band gap of 1.9 eV^12,13^ lies above the ideal range for single-junction photovoltaics, as the Shockley–Queisser limit places the maximum theoretical efficiency at 1.3 to 1.4 eV. Band gap engineering through alloying is therefore a natural strategy to enhance its photovoltaic potential. Partial substitution on the B-site with Ti^14,15^ or on the X-site with Se^16^ reduces the band gap into the optimal range.^13,16–19^ An additional benefit is that Se incorporation is predicted to weaken electron–phonon coupling, potentially suppressing nonradiative recombination in BaZrS_3*x*_Se_3–3*x*_ alloys.^18^ Recent first-principles studies of Ba- and Sr-based zirconium chalcogenide perovskites have likewise demonstrated pronounced differences in electronic and optical properties between the corresponding sulfide and selenide compounds.^20,21^ These studies primarily consider the properties of prescribed crystal structures, whereas the finite-temperature interplay between phase stability, anion ordering, and optical response remains largely unexplored. While alloying thus improves optoelectronic properties, it also modifies bonding and lattice energetics, raising fundamental questions about phase stability and anion ordering.

The structural stability of the perovskite sulfide end member is well established. At ambient conditions, BaZrS_3_ adopts the corner-sharing perovskite structure with orthorhombic *Pnma* symmetry.^22^ Upon heating, it undergoes displacive phase transitions to tetragonal and cubic polymorphs.^23–26^

In contrast, there are conflicting structures reported for the perovskite selenide end member. An early experimental attempt to synthesize stoichiometric BaZrSe_3_ via a solid-state reaction instead yielded the off-stoichiometric columnar compound Ba_15_Zr_14_Se_42_, composed of face-sharing ZrSe_6_ octahedra.^27^ More recently, stoichiometric BaZrSe_3_ in a related face-sharing hexagonal phase with *P*6_3_/*mmc* symmetry has been reported for nanocrystalline samples prepared by hot injection.^28^ First-principles studies constrained to the 1:1:3 stoichiometry instead predict the needle-like δ polymorph with edge-sharing octahedra and *Pnma* symmetry.^29,30^ Se-rich corner-sharing perovskite phases, including stoichiometric BaZrSe_3_, have been realized using nonequilibrium methods.^16,19^ Although these perovskite phases are likely metastable, they exhibit long-term stability at ambient conditions. For the mixed-anion series BaZrS_3*x*_Se_3–3*x*_, powder synthesis studies indicate phase segregation above approximately 40% Se,^9^ also implying limited stability of the perovskite framework at high Se content under near-equilibrium conditions.

Collectively, the available evidence suggests that the perovskite form of BaZrSe_3_ is not the equilibrium ground state under ambient conditions, but that it is kinetically stable. However, a unified thermodynamic description capable of reconciling equilibrium phase segregation, metastable perovskite formation, and possible anion ordering across composition and temperature has not yet been established. Developing such a description is essential for the optimization of mixed-anion chalcogenide perovskites and their optical properties.

Here, we establish the temperature–composition phase behavior of the mixed BaZrS_3*x*_Se_3–3*x*_ system combining atomic scale modeling and scanning transmission electron microscopy (STEM) analysis. Building on our previously developed data set for BaZrS_3_,^26^ we train a machine-learned interatomic potential (MLIP) with density-functional theory (DFT) data. This approach enables large-scale sampling of the configurational and vibrational degrees of freedom while retaining close to hybrid-functional accuracy. Using molecular dynamics (MD) and Monte Carlo molecular dynamics (MCMD) simulations, we explore anion configurations and competing structural motifs over a broad range of temperatures and compositions, keeping the Ba:Zr:(S + Se) ratio fixed at 1:1:3. We predict the emergence of an unusual trans-ordered S/Se arrangement at 33% S content, which we confirm experimentally by STEM. Furthermore, we construct a temperature–composition phase diagram that rationalizes the experimentally observed stability limits of the perovskite phase and clarifies the thermodynamic origins of metastability in Se-rich compositions. Together, these results provide quantitative thermodynamic guidelines for stabilizing Se-rich perovskite phases and tuning anion order to optimize band gap and phase stability in lead-free chalcogenide absorbers. Lastly, we analyze the dielectric function as well as the absorption coefficient, and demonstrate that composition, crystal structure, and anion ordering jointly control the optical band gap.

## Methods

The MLIP was constructed using the neuroevolution potential (NEP) framework^32^ implemented in gpumd,^33^ with reference data obtained from DFT calculations using the HSE06 exchange–correlation functional^34^ and fhi-aims.^35^ MD and combined MCMD simulations^36^ were used to sample structural and anion configurations. The large-scale finite-temperature and free-energy simulations employed supercells containing 8640 to 23,040 atoms, whereas dielectric functions were calculated for snapshots of 40-atom cells. All MD simulations used a time step of 1 fs, with *N*_X_ Monte Carlo trial moves attempted every 100 fs in the MCMD simulations, where *N*_X_ is the number of X-site atoms in the simulation cell. End-member free energies were obtained by thermodynamic integration with respect to an Einstein crystal reference,^37^ and composition-dependent free energies were obtained by integrating chemical-potential differences.

Dielectric functions were calculated using vasp^38^ with the PBE functional^39^ and a rigid scissors correction of 0.63 eV derived from HSE06 band gaps. Optical band gaps were extracted from the resulting absorption coefficients using Tauc analysis.

Experimental anion ordering was examined using atomic-resolution high-angle annular dark-field (HAADF) STEM at 200 kV. Anion-column intensities were extracted using two-dimensional Gaussian fits and compared with simulated images using Moran’s *I* spatial autocorrelation parameter.^40^ Further details on system sizes, equilibration and sampling times, individual simulation protocols, and finite-size convergence are provided in the Supporting Information, together with the detailed computational and experimental procedures.

## Results and Discussion

### Mixing Energies and Ordered Structures

The MLIP developed in this work is based on the NEP framework^32,33,41–44^ (Supporting Note 1) and achieves test-set root-mean-square errors of 4.9 meV atom^–1^ for energies, 0.0843 eV Å^–1^ for forces, and 0.2093 GPa for stresses, with the corresponding parity plots shown in Figure S1. Furthermore, the model accurately reproduces the small energy differences obtained from HSE06 hybrid-functional DFT calculations^34,35^ (Supporting Note 2) between the perovskite, needle-like (δ *Pnma*), and hexagonal (*P*6_3_/*mmc*) phases of the end members BaZrS_3_ and BaZrSe_3_ (Supporting Note 4 and Table S1). In addition, the MLIP yields mixing energies in quantitative agreement with the corresponding DFT results across the full composition range (Supporting Note 5; Figures S2 and S3), demonstrating the reliability of the model for thermodynamic analysis. We note that throughout this study the mixing energy is defined relative to the pure needle-like end members (Supporting Note 8).

The relative 0 K stability of the phases within the 1:1:3 stoichiometry was further assessed using simulated annealing in supercells containing several hundred atoms, which are computationally inaccessible at the DFT level (Supporting Note 5). This analysis identifies the δ needle-like phase as the lowest-energy structure across nearly the entire composition range up to approximately 95% sulfur (Figure 1). These results indicate a two-phase coexistence region separating the perovskite and needle-like phases at low temperature, which we analyze in more detail below (Section Phase Diagram).

**Figure 1:**
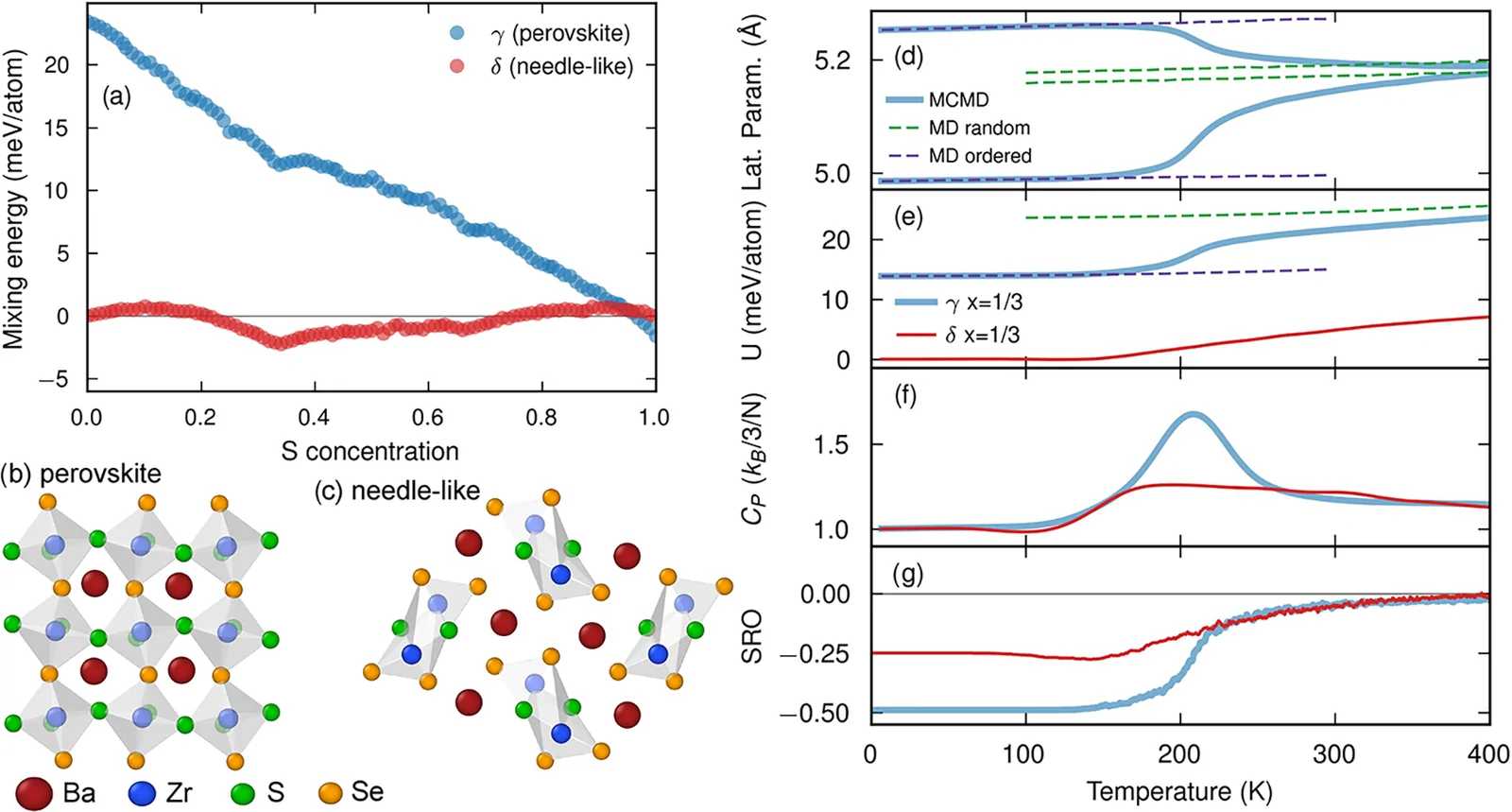
Mixing energies and order–disorder transition from simulations. (a) Comparison of the mixing energies of the perovskite (γ) and needle-like (δ) phases obtained from MCMD cooling simulations. The structures in (b) and (c) display the ordered structures at *x* = 1/3 with Ba atoms shown in red, Zr atoms in blue, S atoms in green, and Se atoms in orange. The Zr-centered coordination polyhedra are shown in gray. Structures were visualized using ovito.^31^ (d–g) Thermodynamic properties from heating simulations at *x* = 1/3 starting from the ordered state. (d) Lattice parameters referred to the primitive cubic cell for the perovskite (γ) phase using both MCMD and MD with fully ordered or fully randomized occupations. (e) Change in potential energy after removal of the Dulong–Petit contribution ( $\frac{3}{2}k_{B}T$ ), (f) heat capacity *C*_P_, and (g) nearest-neighbor Warren–Cowley short-range order (SRO) parameter.

For both the perovskite and needle-like structures, the mixing energy exhibits a pronounced local minimum near *x* = 1/3, corresponding to layered anion ordering patterns commensurate with the respective primitive unit cells (Figure 1b,c). The enhanced stability at *x* = 1/3 can therefore be associated with the commensurability of this composition with the preferred layered ordering motif, allowing the distinct local environments of S and Se to be accommodated periodically without disrupting the ordering pattern. In the perovskite case, this trans-type ordering is analogous to arrangements previously identified in mixed-halide perovskites.^45^ However, such layered ordering appears to be uncommon in oxychalcogenide perovskites; oxysulfide and oxynitride perovskites typically favor cis-configurations, in which identical anions occupy adjacent sites rather than opposite vertices of the octahedron.^46–49^ Studies of oxysulfides further demonstrate that anion ordering can strongly influence electronic properties, underscoring the importance of understanding the origin of the ordered structures identified here^47^ (see Section Optoelectronic Properties).

We propose that layered ordering is energetically favored in BaZrS_3*x*_Se_3–3*x*_ because it enables octahedral tilting to adapt to the local chemical environment. In the orthorhombic *Pnma* structure with tilt pattern *a* ^–^ *a* ^–^ *c* ^+^, layered ordering leads to the *c* ^+^ rotation acting predominantly on Se anions, while the *a* ^–^ tilts involve both S and Se. Consistent with this interpretation, the relaxed Zr–Se–Zr bond angles are smaller than the corresponding Zr–S–Zr angles (155° versus 156°), reflecting a slightly larger tilt amplitude associated with the larger ionic radius of Se. Although layered ordering can induce significant interlayer strain in oxysulfide and oxynitride systems with large ionic-radius mismatch,^47^ the smaller size difference between S and Se likely mitigates such strain here, stabilizing the observed trans-type ordering.

### Order–Disorder Transitions

To assess the finite-temperature ordering behavior of BaZrS_3*x*_Se_3–3*x*_, we performed heating simulations starting from the ordered ground states identified at 0 K (Supporting Note 6 and 7).^33,36,37,50,51^ We focus on the composition *x* = 1/3, which exhibits the strongest ordering tendency in the mixing energies. The temperature evolution of the potential energy, heat capacity, and Warren–Cowley SRO parameter^52^ reveals an order–disorder transition near 210 K for both the δ and γ phases (Figure 1d–g). Finite-size tests using supercells containing 320 to 20 480 atoms show that the temperature dependence of the energy, heat capacity, and SRO parameter is well converged for the 8640-atom cells used for the production simulations (Figure S4). The transition appears broad, consistent with a continuous loss of long-range order rather than a first-order discontinuity. Accordingly, no sharp jump in the energy is observed.

To demonstrate the impact of ordering, we additionally performed MD simulations of the perovskite phase with fully ordered and fully randomized anion occupations (Figure 1d,e). In these simulations, the system remains trapped in the imposed configurations, preventing equilibration between ordered and disordered states. In contrast, the combined MCMD approach enables exchange of S and Se on the X-site sublattice and produces a smooth crossover from the ordered to the disordered regime as temperature increases. Above the transition temperature, the SRO parameter decreases continuously toward zero, reflecting progressive randomization of S and Se on the X-site sublattice. Similar order–disorder behavior is observed across the composition range, but the transition is particularly pronounced at *x* = 1/3, where ideal commensurate ordering patterns are possible.

To provide experimental evidence for this unusual anion ordering and its associated order–disorder transition, we carried out STEM characterization of BaZrS_3*x*_Se_3–3*x*_ thin-film cross sections with compositions near *x* = 1/3 (Supporting Note 10–12).^12,19^ Atomic-number-sensitive HAADF imaging along the pseudocubic ⟨100⟩ direction reveals an atom-column arrangement consistent with the perovskite structure of BaZrS_3*x*_Se_3–3*x*_ (Figure 2a,b). Significant intensity variations are observed at the anion columns, with columns along projected ⟨110⟩ directions exhibiting alternating low and high intensity. To quantify these variations, peak intensities extracted from two-dimensional Gaussian fits were mapped onto the projected structure (Figure 2c), revealing spatial patterns consistent with layered anion ordering.

**Figure 2:**
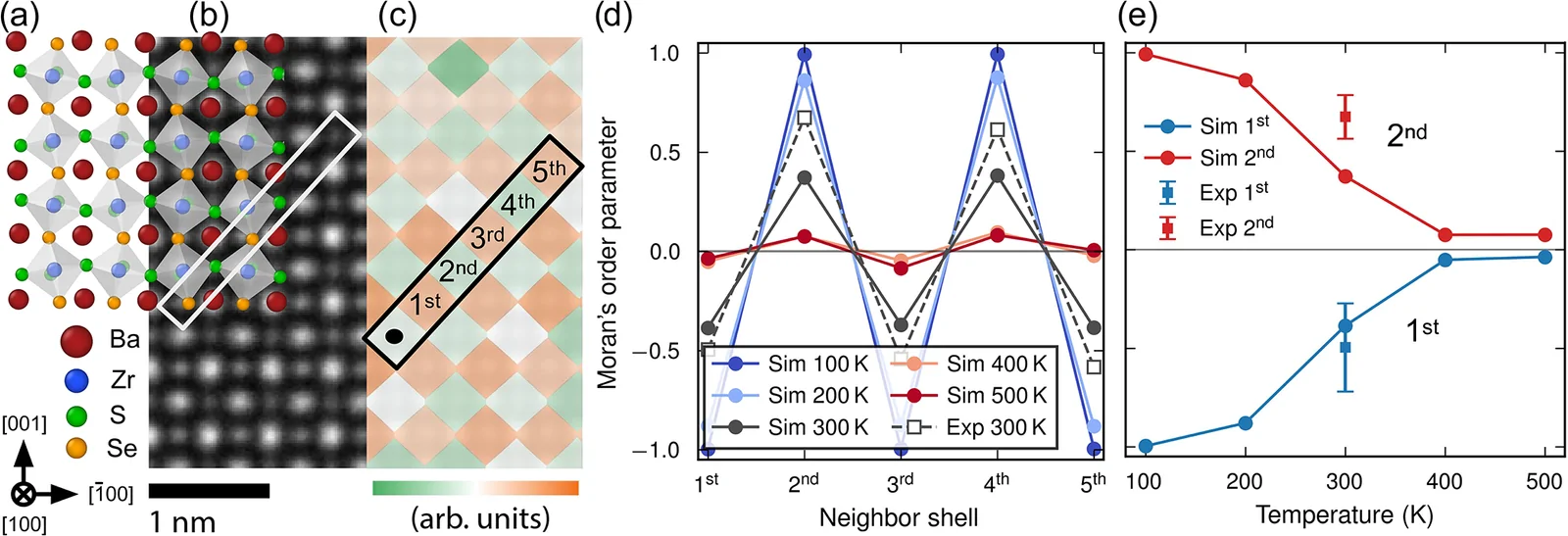
Experimental observation of ordering. (a) Atomistic structure of the ideal ordered perovskite at *x* = 1/3. (b) STEM HAADF image of a sample of BaZrS_3*x*_Se_3–3*x*_ with *x* ≈ 0.26 along the pseudocubic ⟨100⟩ zone axis, overlaid with the ideal atomic structure. (c) Anion atom column intensities corresponding to the X-sites extracted from the HAADF images (where orange and green markers scale with higher (Se) and lower (S) image intensity, respectively). (d) Moran’s *I* order parameter from experiment at approximately 300 K (Exp) and from images based on the atomistic simulations (Sim) as a function of increasing pseudocubic ⟨110⟩ neighbor shell. (e) Moran’s order parameter as a function of temperature for the first and second neighbor shell.

To enable a direct comparison between experiment and simulation, we evaluated the Moran’s *I* nearest-neighbor autocorrelation statistic for both experimental images and simulated STEM images generated from MCMD snapshots (Figure 2d,e; see eq (9)).^40^ As a function of increasing anion neighbor shell along ⟨110⟩ directions, the order parameters extracted from simulated images exhibit an order–disorder transition consistent with that obtained directly from the atomistic simulations. The experimentally determined order parameters at approximately 300 K fall between the simulated values at 200 and 300 K, indicating partial ordering at room temperature. These results confirm that the anion ordering predicted by simulations is observed experimentally at comparable temperature and composition.

It is important to note that the perovskite γ phase is thermodynamically metastable with respect to the needle-like δ phase within the 1:1:3 stoichiometry, while its experimental persistence at room temperature suggests kinetic stabilization against transformation to the equilibrium structure. The persistence of nonzero SRO above the nominal transition temperature, including at room temperature, suggests that local anion correlations can survive even after long-range order is lost.

### Phase Diagram

We now examine the temperature–composition phase behavior of BaZrS_3*x*_Se_3–3*x*_. We first determine the transitions among the perovskite polymorphs from MCMD heating simulations (Figure S5), which reveal successive transitions from the orthorhombic γ (*Pnma*) to the tetragonal β (*I*4/*mcm*) and finally to the cubic α (*Pm*3̅*m*) phase across the composition range. These transitions are identified through a combined analysis of lattice parameters, heat capacities, and symmetry-adapted octahedral tilt mode projections^53^ as functions of temperature. For the BaZrS_3_ end member, the predicted orthorhombic-to-tetragonal transition at approximately 690 K is in reasonable quantitative agreement with temperature-dependent X-ray diffraction studies, which identify structural changes toward higher-symmetry phases in the approximately 700 to 800 K range.^23–25^

In contrast, the transition between the needle-like δ phase and the perovskite γ phase is not directly accessible from MD or MCMD simulations on the simulated time scales, consistent with a substantial kinetic barrier separating these structurally distinct phases. We therefore determine this boundary from free energy calculations (Supporting Note 7).

We begin by computing the Gibbs free energy of the pure BaZrSe_3_ end member, *G*(*x* = 0, *T*), using thermodynamic integration to an Einstein crystal reference (Figure 3a). The δ phase is stable up to approximately 870 K, above which the γ phase becomes thermodynamically favored. Although the γ phase is higher in energy by about 24 meV atom^–1^ at 0 K (Table S1), it possesses a significantly larger vibrational entropy, which stabilizes it at elevated temperature. This enhanced entropy arises from a greater density of low-frequency phonon modes in the γ phase (Figures 3a and S6).

**Figure 3:**
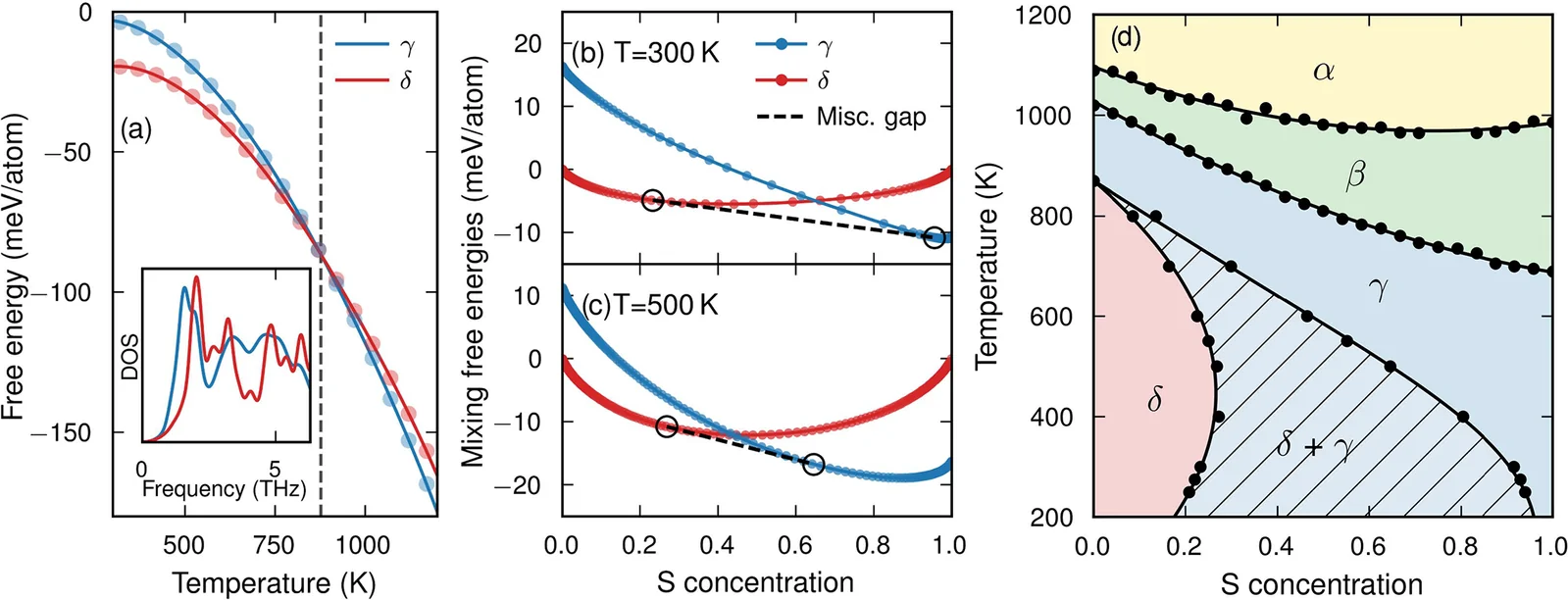
Free energies and phase diagram. (a) Free energies of the pure Se (*x* = 0) γ and δ phases. The inset shows the vibrational density of states obtained from MD at 300 K via the mass-weighted velocity autocorrelation function. Mixing free energies for γ and δ phases at (b) 300 K and (c) 500 K. The dashed lines indicate the miscibility gap with end points being marked with open circles. (d) Predicted phase diagram for the BaZrS_3*x*_Se_3–3*x*_ system. Here, α (*Pm*3̅*m*), β (*I*4/*mcm*), and γ (*Pnma*) refer to the three perovskite phases, while δ refers to the needle-like phase (*Pnma*). The striped δ + γ region indicates the two-phase region.

Using *G*(*x* = 0, *T*) as a reference, the mixing free energy Δ*G*_mix_(*x*, *T*) is obtained across the full composition range via free energy integration (Supporting Note 8). At 300 K, a two-phase region is predicted between approximately *x* = 0.2 and *x* = 0.95, separating the γ and δ phases (Figure 3b). At 500 K, this coexistence region narrows and shifts asymmetrically to approximately *x* = 0.25 and *x* = 0.65 (Figure 3c).

Free energy calculations performed over a dense grid of temperatures then yield the full temperature–composition phase diagram of BaZrS_3*x*_Se_3–3*x*_ (Figure 3d). On the Se-rich side, the needle-like δ phase is thermodynamically stable, whereas on the S-rich side the perovskite phase is favored, with a two-phase coexistence region at intermediate compositions. At higher temperatures, the orthorhombic γ phase is stabilized across the entire composition range and subsequently transforms to the tetragonal β and cubic α polymorphs upon further heating.

When synthesized via a conventional solid-state reaction at 700 K,^9^ Se substitution up to approximately 40% in the sulfide perovskite has been reported without observation of nonperovskite precipitates at room temperature. Our calculations predict that only about 5% Se is thermodynamically soluble in the perovskite phase at 300 K. However, the calculated solubility limit increases to approximately 20% at 400 K and to 35% at 500 K, bringing the theoretical predictions closer to experimental observations under elevated-temperature growth conditions. Furthermore, the computed free energy differences are small, and modest uncertainties in the underlying DFT reference data, the MLIP model, or the thermodynamic sampling can shift phase boundaries. Indeed, first-principles predictions of phase transition temperatures in similar systems can commonly differ from experiment by 100 to 200 K depending on the underlying exchange-correlation functional.^26,54^ This range should be regarded as an indication of the expected scale of uncertainty rather than as a formal error bar for the present system. The experimental persistence of a mixed perovskite phase at room temperature, despite the predicted thermodynamic driving force for phase separation involving the δ phase, suggests that kinetic limitations hinder the structural transformation and phase separation.

### Optoelectronic Properties

Finally, to understand how the electronic and optical properties of the material are affected by changes in composition, phase, and atomic ordering, we compute the dielectric function and the absorption coefficient. The dielectric functions are calculated at the PBE level, with a rigid scissors correction of 0.63 eV derived from HSE06 (α = 0.25) band gaps applied to correct the absorption energies. The role of spin–orbit coupling (SOC) is assessed separately using HSE06 + SOC calculations for the fully relaxed S and Se end members, where it decreases the band gap by up to 0.17 eV (see Supporting Note 3 for further details).

The absorption coefficients of the perovskite and needle-like phases at 300 K are calculated for all compositions (Figure 4a,b). For both phases, the onset of absorption shifts monotonically to higher energies with increasing sulfur concentration. Notably, the needle phase exhibits an extended but weak absorption tail, in contrast to the sharper onset observed for the perovskite phase. A similar difference between the two phases has previously been reported for SrZrS_3_.^55^

**Figure 4:**
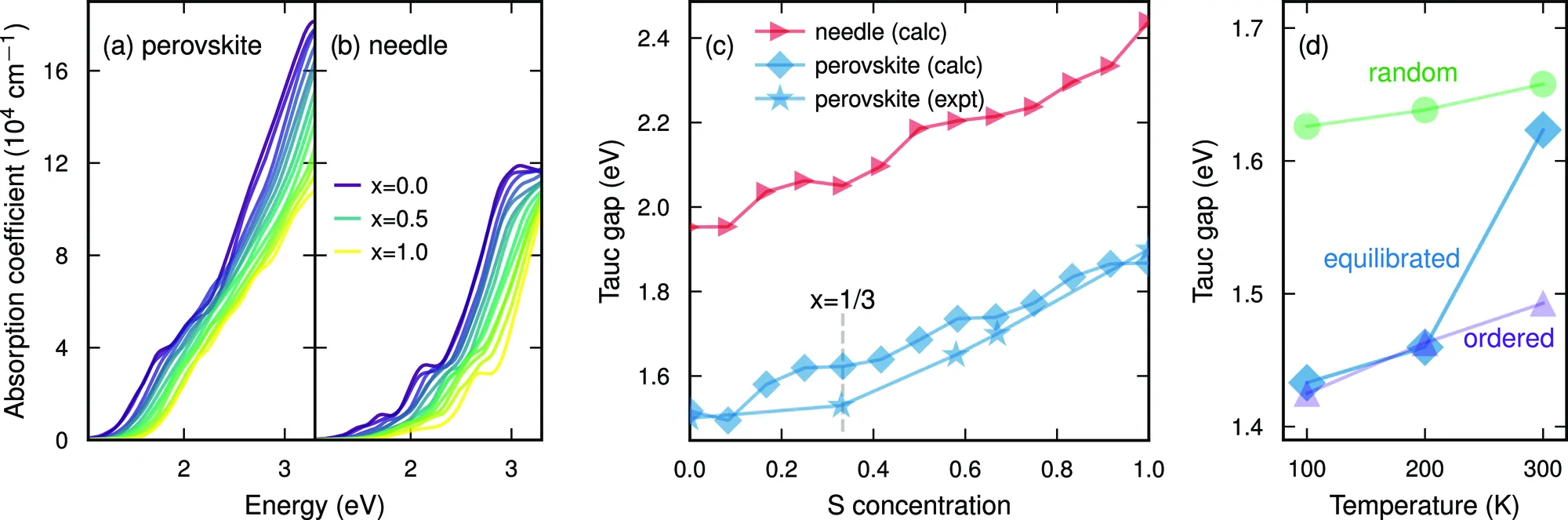
Optical properties. (a, b) Absorption coefficients of the perovskite and needle-like phases, respectively, at 300 K for varying sulfur concentrations using equilibrated MCMD snapshots. (c) Tauc gaps obtained from the data in (a, b) (calculated), together with experimental data from ref 16. (d) Tauc gaps at 33% sulfur concentration obtained from three different simulations: MD with random occupation, MD with ordered occupation, and fully equilibrated MCMD at 100, 200, and 300 K.

From the absorption coefficients, we extract the optical band gap via a Tauc analysis (Figure 4c; see Supporting Note 3 and Figure S8 for details). For both phases, the Tauc gap increases monotonically with increasing sulfur content, in agreement with previous experimental and theoretical studies.^16–18^ The predictions are in very good agreement with experimental data from ref 16, particularly for the end members. Note that excitonic electron–hole interactions are not explicitly included in the present calculations and may affect the absolute near-edge absorption and optical gaps, although the focus here is primarily on relative changes with composition, phase, and anion ordering.

Previous DFT studies have reported that the needle phase exhibits a lower *fundamental* band gap than the perovskite phase.^56^ Consistently, the band gaps obtained directly from the electronic eigenstates are smaller for the needle phase than for the perovskite phase (Table S2). In contrast, the Tauc analysis shows that the *optical* gap of the needle phase is notably larger. This discrepancy arises from the extended but weak absorption tail observed in the needle phase (Figure 4b), highlighting that the fundamental and optical gaps can differ significantly.

Finally, we demonstrate that the anion ordering identified in this work (Figure 2) has a significant impact on the optical properties. To this end, we perform three types of simulations at 33% S and temperatures of 100, 200, and 300 K: MD with fully ordered anion occupation, MD with random occupation, and fully equilibrated MCMD. Since the optical properties are evaluated from finite-temperature MD snapshots using the corresponding temperature-dependent lattice parameters, thermal expansion and vibrational structural fluctuations are included in these calculations. Analysis of the optical properties from MD simulations with fixed occupations (ordered or random) reveals a consistent difference of 0.16 to 0.19 eV in the Tauc gap, independent of temperature. In both cases, the Tauc gap increases with temperature, opposite to typical semiconductor behavior but consistent with trends reported for other perovskites.^57^ However, this variation is considerably smaller than the rapid change exhibited by the fully equilibrated system across the order–disorder transition. The observed difference is therefore primarily attributable to the ordering of S and Se atoms in the system.

## Conclusions

By combining a MLIP with large-scale MD simulations, we have revealed ordering phenomena in the BaZrS_3*x*_Se_3–3*x*_ perovskite system. The simulations predict the emergence of a trans-ordered configuration with alternating layers of S and Se atoms, which is particularly pronounced at *x* = 1/3.

This ordering is supported by STEM measurements and corresponding image simulations. In addition, we performed free-energy calculations based on MCMD simulations to construct the full phase diagram, considering all relevant ABX_3_ polymorphs. Our results show that while the perovskite phase is thermodynamically stable only at high sulfur content, a wide two-phase region exists, and the perovskite phase remains dynamically stable across the entire composition range. Finally, we demonstrate that composition, crystal phase, and anion ordering all strongly influence the optoelectronic properties of this prototypical chalcogenide perovskite. In particular, the broad order–disorder transition centered near 210 K, with partial ordering persisting to room temperature, leads to a shift in the Tauc gap of approximately 0.16 to 0.19 eV. This demonstrates that a detailed understanding of the underlying thermodynamic properties, such as phase stability and ordering, is essential for the rational design of materials with tailored properties.

## Supplementary Material



## Data Availability

The NEP model and DFT data generated in this study are openly available via Zenodo at 10.5281/zenodo.19483979.

## Notes

Additional computational and experimental methods; MLIP construction and validation; dielectric-function calculations; mixing-energy, free-energy, and phase-diagram methodology; finite-size convergence and order-parameter analyses; film-growth and STEM methods; supplementary figures and tables (PDF)
