# Supplementary material for: Anion Ordering and Phase Stability Govern Optical Band Gaps in BaZrS3x Se3–3x
Source: Chem Mater. 2026 Sep 22;38(19):10251–8. doi: 10.1021/acs.chemmater.6c01949 (PMC13673944; doi:10.1021/acs.chemmater.6c01949)
Supplement: Supplementary file 1 [file cm6c01949_si_001.pdf]

Supporting Information

Anion Ordering and Phase Stability Govern Optical Band Gaps in $BaZrS_{3x}Se_{3-3x}$

Erik Fransson,[1] Michael Xu,[2] Prakriti Kayastha,[3,4] Kevin Ye,[2] Ida Sadeghi,[2] Rafael Jaramillo,[2] James M. LeBeau,[2] Lucy Whalley,[3] and Paul Erhart[1,*]

[1] Department of Physics and Astronomy, Chalmers University of Technology, SE-41296, Gothenburg, Sweden
[2] Department of Materials Science and Engineering, Massachusetts Institute of Technology, Cambridge, MA 02139, United States of America
[3] School of Engineering, Physics and Mathematics, Northumbria University, Newcastle upon Tyne, NE1 8QH, United Kingdom
[4] Department of Chemistry, University College London, London WC1E 6BT, United Kingdom
[*] erhart@chalmers.se

Contents

Supplementary Notes **S2**
SN1. Neuroevolution potential construction . . . S2
SN2. Density functional theory calculations . . . S2
SN3. Dielectric function calculations . . . S2
SN4. Ground state structures . . . S3
SN5. Mixing energies . . . S3
SN6. Molecular dynamics and free energy calculations . . . S3
SN7. Monte Carlo–molecular dynamics . . . S3
SN8. Free energy and phase diagram construction . . . S4
SN9. Order parameters . . . S4
SN10. Film growth . . . S4
SN11. Scanning transmission electron microscopy . . . S5
SN12. Scanning transmission electron microscopy image analysis . . . S5

Supplementary Figures **S6**
S2. Mixing energies from MLIP and DFT . . . S6
S3. Mixing energies for different phases . . . S7
S4. System-size convergence of the order–disorder transition . . . S7
S5. MCMD simulations . . . S8
S6. Phonon dispersions . . . S8
S7. PBE-to-HSE06 band-gap correction . . . S9
S8. Tauc gap fitting . . . S9

Supplementary Tables **S10**
S1. Energy differences . . . S10

Supplementary References **S11**

Supplementary Notes

Supplementary Note 1: Neuroevolution potential construction

A machine-learned interatomic potential (MLIP) was constructed using the neuroevolution potential (NEP) framework as implemented in GPUMD version 3.9.5 (1, 2). The NEP formalism was chosen for its high computational efficiency relative to other state-of-the-art MLIP approaches, which is essential for the extensive configurational and thermodynamic sampling performed here. Its local descriptor-based architecture additionally enables efficient local Monte Carlo updates, providing a substantial computational advantage for hybrid Monte Carlo molecular dynamics (MCMD) simulations (Note 7).

Training structures were generated from the relevant structural prototypes, including corner-sharing perovskites ($Pnma$, $I4/mcm$, $Pm\bar{3}m$), a needle-like edge-sharing phase ($Pnma$), and the hexagonal phase ($P6_3/mmc$) using the ASE (3) and HIPHIVE (4) packages. Mixed-anion configurations were generated by enumeration using ICET (5) following the algorithm of Hart and Forcade (6), complemented by randomly occupied supercells. The training set was iteratively refined by adding structures generated via molecular dynamics (MD) and MCMD simulations in the NPT ensemble between 0 and 1200 K. About 100 selected structures from the $BaZrS_3$ training dataset of Ref. 7 were also incorporated. The final dataset contained 2838 structures. The NEP model used the NEP4 formalism with radial and angular cutoffs of 8 Å and 4 Å, respectively, with $n_{\mathrm{max}} = 4$ and a basis size of 8 for both radial and angular descriptors. The maximum angular orders for the three-, four-, and five-body descriptors were $l_{\mathrm{max}} = 4$, 2, and 0, respectively. The neural network contained a single hidden layer with 30 neurons, corresponding to a 30–30–1 architecture and a total of 5281 optimized model parameters. Training employed $\lambda_1 = \lambda_2 = 0.005$, relative energy, force, and virial weights of 1, 1, and 0.1, respectively, and was carried out for a total of 200 000 generations.

Model validation yields train (test) energy root-mean-square error (RMSE) values of 0.0034 eV/atom (0.0049 eV/atom), a force RMSE of 0.0835 eV/Å (0.0843 eV/Å), and a stress RMSE of 0.2058 GPa (0.2093 GPa). The training and test subsets were generated using a k-fold split with $k = 10$. The final model used throughout the paper was subsequently trained on the full dataset.

The ASE (3) and CALORINE (8) packages were used to prepare the training structures, set up MD simulations and post-process the results. Random displacements were generated using the HIPHIVE package (4).

The complete density-functional theory (DFT) dataset and trained NEP model used in this work are openly available at `https://doi.org/10.5281/zenodo.19483979`.

Supplementary Note 2: Density functional theory calculations

The DFT calculations used to generate the MLIP reference data and evaluate phase energetics were performed with the FHI-AIMS code (9) using the HSE06 exchange–correlation functional (10). All computational parameters were identical to those reported in Ref. 7. A Monkhorst–Pack k-point mesh with a minimum k-spacing of 0.2/Å was employed. The *light* basis set was used throughout. Stress tensors were evaluated for small and medium-sized cells included in the training set, but were not computed for structures containing more than approximately 50 atoms due to memory constraints.

Supplementary Note 3: Dielectric function calculations

Dielectric functions were calculated using VASP (11) with the PBE functional (12), using projector-augmented wave (13, 14) setups. We use a plane-wave energy cutoff of 520 eV and automatically generated **k**-point grids with a maximum spacing of 0.2/Å.

The dielectric function $\epsilon(\omega) = \epsilon_1(\omega) + i\epsilon_2(\omega)$ is converted to the absorption coefficient via

$$\alpha(\omega) = \frac{\sqrt{2}\,\omega}{c}\sqrt{\sqrt{\epsilon_1(\omega)^2 + \epsilon_2(\omega)^2} - \epsilon_1(\omega)}. \tag{1}$$

The Tauc gap is subsequently extracted from the absorption coefficient by plotting $(\alpha(\omega)\cdot\hbar\omega)^2$ as a function of photon energy and performing a linear fit to the onset region, excluding the low-energy Urbach tail (Figure S8). The optical band gap (Tauc gap) is taken as the zero-energy intercept of this linear fit.

For each composition and temperature, 5 to 10 snapshots extracted from NVT MCMD and MD simulations of 40-atom cells are used, with lattice parameters fixed to those obtained from the corresponding NPT runs. The

dielectric functions are averaged over these snapshots for each composition and phase. Explicit electron–hole interactions and the resulting excitonic effects are not included in the calculated dielectric functions.

To correct for the band gap underestimation of PBE, we compute HSE06 band gaps for a representative set of snapshots using FHI-AIMS, obtaining a consistent PBE-to-HSE06 shift of 0.63 eV, which is applied as a rigid scissors correction to all dielectric functions. The dielectric functions themselves are therefore calculated at the PBE level and are not recalculated using HSE06.

Additionally, we assess the role of spin-orbit coupling (SOC) by computing HSE06+SOC band gaps for the fully relaxed pure S and Se end-members in both the perovskite and needle (both *Pnma*) phases; results are provided in Table S2. SOC reduces the band gap in both cases, with a larger effect for Se than for S, consistent with the expected trend for heavier chalcogenides (15).

Supplementary Note 4: Ground state structures

For the sulfide, the perovskite γ (*Pnma*) phase is identified as the ground state, in agreement with experiment, with the non-perovskite δ phase lying 2 meV/atom higher in energy. The hexagonal $P6_3/mmc$ phase is substantially higher in energy, by 72 meV/atom. For the selenide, the non-perovskite δ phase is identified as the ground state, with the perovskite γ phase 24 meV/atom higher in energy (see Table S1). Again, the hexagonal $P6_3/mmc$ phase is much higher in energy at 33 meV/atom, and remains energetically unfavorable across the full composition range. Accordingly, this hexagonal phase is excluded from further thermodynamic analysis.

Supplementary Note 5: Mixing energies

Mixing energies at 0 K were computed for relaxed structures generated by enumeration of symmetry-inequivalent configurations based on the parent perovskite and δ needle-like phases, as well as from cooling MCMD simulations (Figure S2 and Figure S3). The cooling simulations were performed from 1200 K to 0 K over 40 ns using supercells containing approximately 300 to 400 atoms. For nearly all compositions, the cooling simulations yield structures with energies equal to or lower than those obtained from enumeration. The relaxed enumerated structures themselves remain in quantitative agreement with direct DFT calculations, confirming the reliability of the NEP model.

Supplementary Note 6: Molecular dynamics and free energy calculations

All MD simulations employed a timestep of 1 fs. Constant-pressure simulations were performed in the NPT ensemble using the stochastic cell rescaling barostat with a pressure target of 0 GPa. The heating and cooling simulations employed supercells containing 20 480 atoms. Equilibrium lattice parameters at each temperature and composition were obtained by averaging over heating and cooling runs.

The Helmholtz free energies of the end members ($x = 0$ and $x = 1$) were computed using thermodynamic integration (TI) with respect to an Einstein crystal reference (16), as implemented in GPUMD (2). The interpolated Hamiltonian was defined as

$$H(\lambda) = (1 - \lambda)H_{\mathrm{Ein}} + \lambda H_{\mathrm{NEP}}, \tag{2}$$

where λ is the Kirkwood coupling parameter (17). The free energy difference between the NEP system and the Einstein crystal reference is given by

$$G_{\mathrm{NEP}} - G_{\mathrm{Ein}} = \int_0^1 \left\langle \frac{\partial H(\lambda)}{\partial \lambda} \right\rangle_\lambda \mathrm{d}\lambda, \tag{3}$$

where $\langle \cdots \rangle_\lambda$ denotes an ensemble average evaluated with the Hamiltonian $H(\lambda)$. TI simulations were performed for 0.1 ns using a spring constant of $4\,\mathrm{eV/Å}^2$ and a supercell containing 23 040 atoms, and the resulting free energies were averaged over 10 independent runs performed under identical conditions.

Supplementary Note 7: Monte Carlo–molecular dynamics

To explicitly sample both vibrational and chemical configurational degrees of freedom in the mixed system, we performed MCMD simulations (18–20), in which MD steps are interleaved with Monte Carlo (MC) trial moves. After every 100 fs of MD, N_{X} MC trial moves were attempted, where N_{X} denotes the number of X-site atoms in the simulation cell. All MD segments employed a timestep of 1 fs. The constant-temperature semi-grand canonical (SGC) simulations employed supercells containing 8640 atoms. Each simulation was equilibrated for 200 ps, after which thermodynamic observables were sampled for approximately 1.8 ns.

Configurational sampling was carried out using flip trial moves in the SGC ensemble, in which the chemical species of a randomly selected X-site atom is changed from S to Se or vice versa. For a binary sublattice,

we define the concentration of species A as $x_\mathrm{A} = N_\mathrm{A}/N$, where N_A is the number of A atoms on the X-site sublattice and N is the total number of X-site atoms.

In the SGC simulations, the chemical potential of species B is taken as the reference, $\mu_\mathrm{B} = 0$, such that μ_A corresponds to the chemical-potential difference $\mu_\mathrm{A} - \mu_\mathrm{B}$. At fixed total number of X-site atoms, this relative chemical potential can equivalently be expressed in terms of the free energy per X-site atom, $f = F/N$, as

$$\mu_\mathrm{A} = \frac{\partial f}{\partial x_\mathrm{A}}. \tag{4}$$

The concentration-dependent free energy $f(x_\mathrm{A}, T)$ can therefore be obtained by thermodynamic integration along the composition axis,

$$f(x_\mathrm{A}, T) = f(0, T) + \int_0^{x_\mathrm{A}} \mu_\mathrm{A}(x'_\mathrm{A}, T)\, \mathrm{d}x'_\mathrm{A}. \tag{5}$$

Supplementary Note 8: Free energy and phase diagram construction

For each structural phase, the Helmholtz free energy $F(x, T)$ is determined as described above, where here and in the remainder of this paper x denotes the S concentration on the X-site sublattice, while the Ba and Zr sublattices remain fixed. We define the mixing free energy of a given phase as

$$\Delta G_\mathrm{mix}(x, T) = F(x, T) - \left[x G_\delta(1, T) + (1 - x) G_\delta(0, T)\right], \tag{6}$$

where $G_\delta(0, T)$ and $G_\delta(1, T)$ are the free energies of the pure Se and pure S needle-like δ phases, respectively. The needle-like phase thus serves as a common reference state for all compositions. Because all calculations are performed at a pressure of 0 GPa, the pV contribution vanishes and $G = F$; we therefore use G and F interchangeably in the following.

Part of the temperature–composition phase diagram is constructed by mapping the convex hull of the free energy as a function of composition at each temperature. Phase coexistence regions are identified from common-tangent constructions, while single-phase stability corresponds to compositions lying on the convex envelope.

In addition to compositional phase separation, the perovskite phase exhibits a continuous tetragonal-to-cubic ($Pm\bar{3}m$) transition. Because this second-order transition does not involve a free-energy crossing between distinct phases, its location cannot be determined from convex-hull analysis. Instead, it is identified from cooling simulations initiated in the cubic phase and monitoring changes in structural and thermodynamic observables, following the procedure of Refs. 7, 21.

Supplementary Note 9: Order parameters

To distinguish the cubic, tetragonal, and orthorhombic perovskite polymorphs observed in the MD and MCMD simulations, we project the instantaneous atomic displacements onto normal modes obtained for the ideal cubic structure corresponding to octahedral tilting distortions (21). Specifically, we evaluate the projection onto the lowest-frequency phonon modes at the M and R points of the Brillouin zone, which correspond to in-phase and out-of-phase octahedral tilts, respectively. The relative amplitudes of these mode projections allow us to identify the tilt patterns characteristic of the cubic, tetragonal, and orthorhombic phases, as discussed in detail in Ref. 7.

Chemical short-range order (SRO) on the X-site sublattice is quantified using the Warren–Cowley parameter (22). For an atom i of type A on the X sublattice, the first-shell parameter is defined as

$$\alpha_i = 1 - \frac{Z_\mathrm{B}}{Z_\mathrm{tot} c_\mathrm{B}}, \tag{7}$$

where Z_B is the number of first-nearest-neighbor B atoms on the X sublattice, Z_tot is the total number of first-nearest neighbors, and c_B is the overall concentration of species B. The SRO parameter is averaged over all X-site atoms and over equilibrated snapshots of the simulation trajectory. Only first-nearest neighbors on the X sublattice are considered. For a random distribution, $\alpha = 0$; $\alpha > 0$ indicates a tendency toward clustering or phase separation, while $\alpha < 0$ signifies preferential unlike-neighbor bonding and chemical ordering.

Supplementary Note 10: Film growth

Thin films were grown in a custom-built chalcogenide molecular beam epitaxy (MBE) system (Mantis Deposition M500). Detailed deposition procedures are described in Refs. 23, 24. Films were deposited on $10\,\mathrm{mm} \times 10\,\mathrm{mm} \times$

0.5 mm $(001)_{PC}$-oriented $LaAlO_3$ substrates (MTI Corporation). Here, PC denotes the pseudocubic setting, and the $(001)_{PC}$ orientation corresponds to the (012) family of reflections in the rhombohedral basis.

Epitaxial $BaZrS_3$ films were first grown and subsequently subjected to selenizing anneals for anion exchange. Prior to growth, substrates were outgassed in the MBE chamber at 1000 °C under flowing H_2S. $BaZrS_3$ films were deposited at 900 °C to 1000 °C using elemental Ba and Zr sources under a 0.8 SCCM flow of H_2S. Alloying with selenium was achieved by annealing the epitaxial $BaZrS_3$ films at 800 °C for 60 min under a mixed chalcogen flow of 0.2 SCCM H_2S and 0.2 SCCM H_2Se.

Cross-sectional compositions were quantified by scanning transmission electron microscopy (STEM) energy-dispersive X-ray spectroscopy. Elemental mapping confirms a uniform distribution of Ba, Zr, S, and Se across the film thickness, yielding an overall composition of $BaZrS_{3x}Se_{3-3x}$ with $x = 0.27$ (23).

Supplementary Note 11: Scanning transmission electron microscopy

Samples for STEM were prepared by non-aqueous mechanical polishing followed by thinning to electron transparency using single-sector argon-ion milling (Fischione 1051 TEM Mill) (25). Atomic-resolution high-angle annular dark-field (HAADF) image series were acquired on a probe aberration-corrected Thermo Fisher Scientific Themis Z S/TEM operated at 200 kV. A probe semi-convergence angle of 18.9 mrad, a detector collection range of 65 mrad to 200 mrad, and a dwell time of 1 µs were used. Image series were aligned and corrected for distortions arising from linear drift using the revolving STEM method (26).

Supplementary Note 12: Scanning transmission electron microscopy image analysis

STEM image simulations were performed using a custom Python implementation of the multislice method (27, 28). For both simulated and experimental HAADF images, peak intensities of anion atomic columns were extracted by fitting two-dimensional Gaussian functions to the projected columns.

To quantify spatial ordering and directly compare simulations with experiment, Moran's I spatial autocorrelation parameter was evaluated for the anion peak intensities (29). For a given feature A (here, the anion column intensity) over N atomic columns, the autocorrelation metric is defined as

$$M^I = \frac{N}{W} \frac{\sum_{i=1}^{N} \sum_{j=1}^{N} w_{ij}(A_i - \bar{A})(A_j - \bar{A})}{\sum_{i=1}^{N}(A_i - \bar{A})^2}, \tag{8}$$

with

$$W = \sum_{i=1}^{N} \sum_{j=1}^{N} w_{ij}, \tag{9}$$

$\bar{A}$ is the mean value of A, and w_{ij} is a weighting factor defining the neighbor relationship between atomic columns i and j. In this work, neighbors are defined along the projected pseudocubic $\langle 110 \rangle$ directions. This metric provides a quantitative measure of spatial correlations in anion intensity variations associated with chemical ordering.

Supplementary Figures

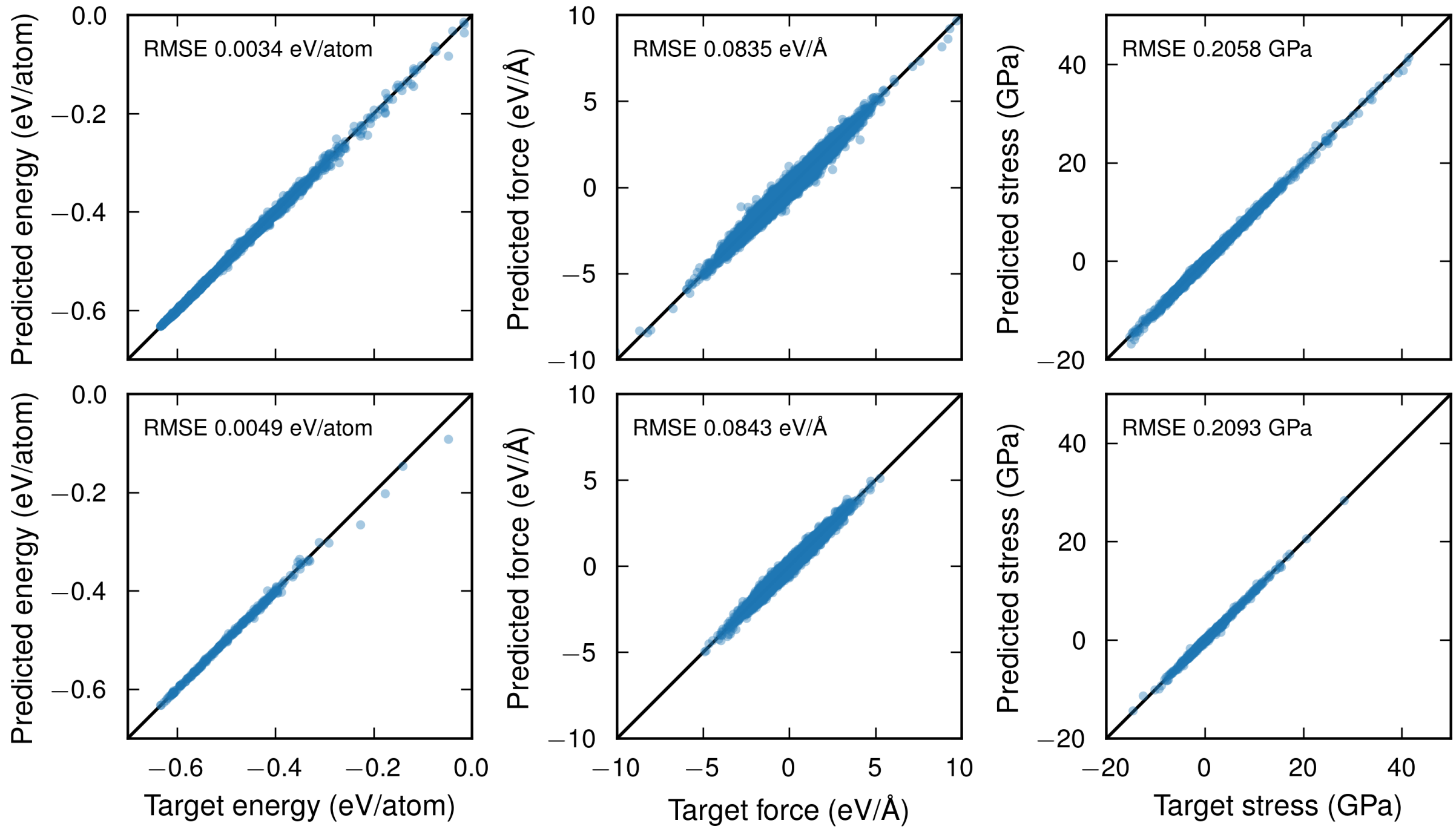

Figure S1: Parity plots for the MLIP compared to the reference DFT data. The top row corresponds to the training data and the bottom row to the test data.

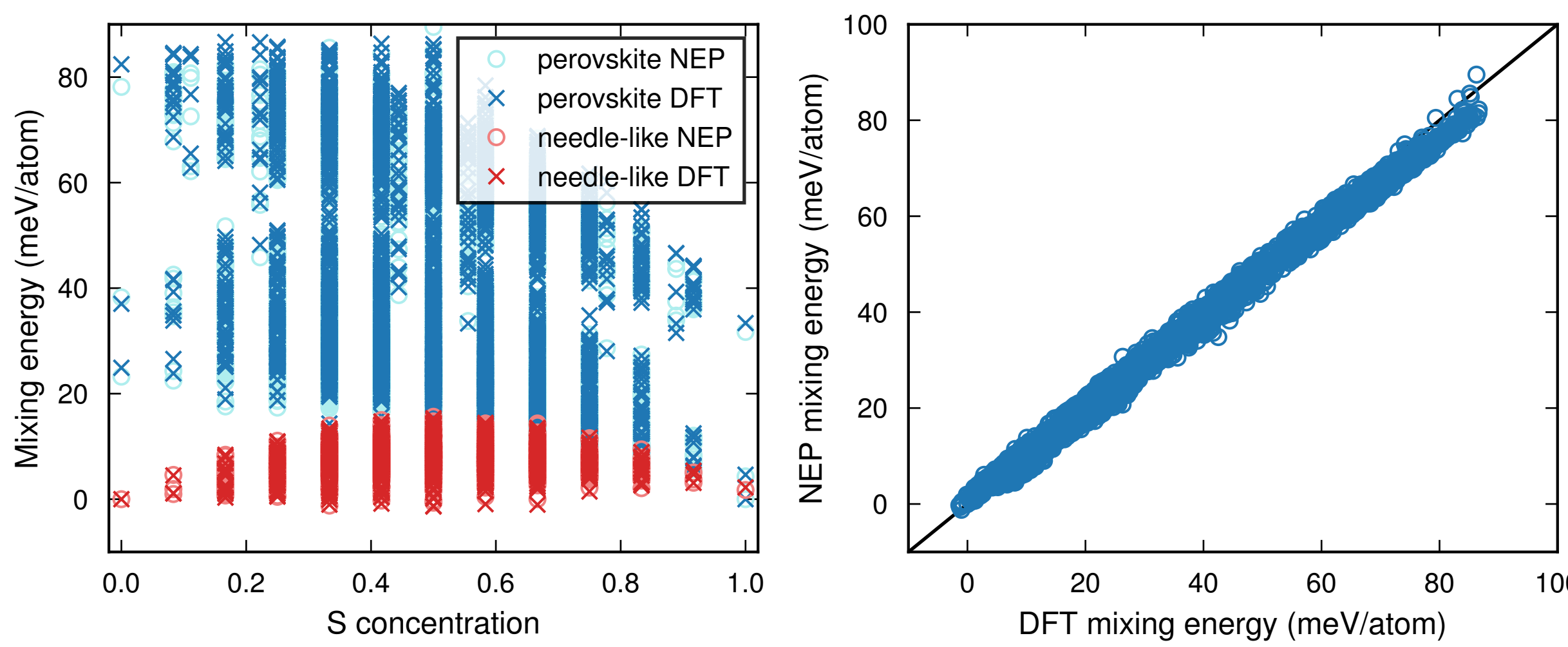

Figure S2: Mixing energies from MLIP and DFT. Left: Mixing energies based on enumerated structures for the perovskite and needle-like (δ) phases calculated with DFT and NEP. Right: Parity plot for the mixing energies.

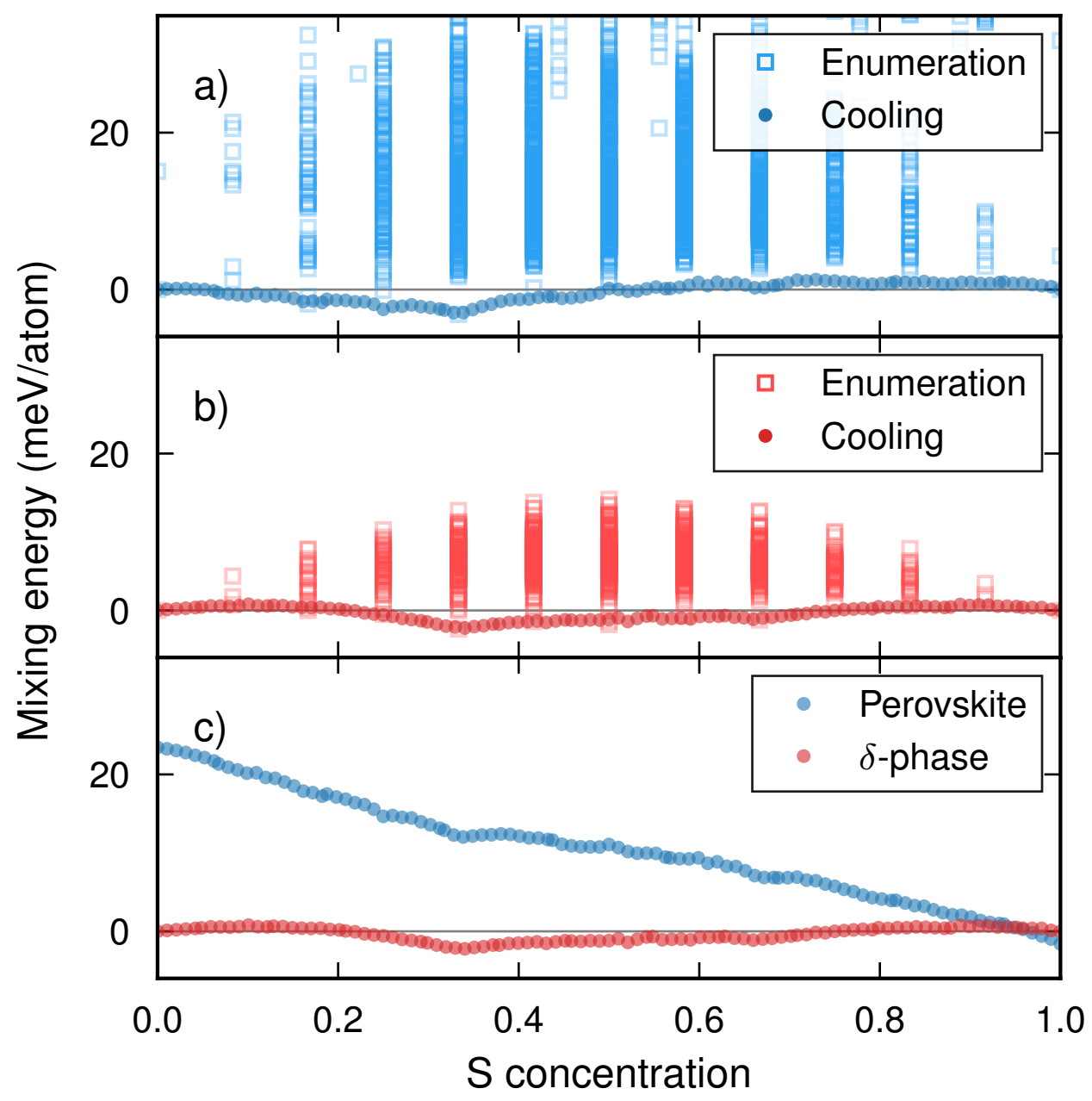

Figure S3: Mixing energy of the (a) perovskite and (b) needle-like (δ) phase as a function of composition. Structures labeled "enumeration" were obtained from systematic combinatorial sampling followed by relaxation, whereas structures labeled "cooling" were obtained from MCMD cooling simulations. The mixing energies are referenced to respective end members of the same structure to facilitate comparison within the respective structure family. (c) Comparison of mixing energies of the perovskite (γ) and needle-like (δ) phases obtained from cooling simulations using a common energy reference.

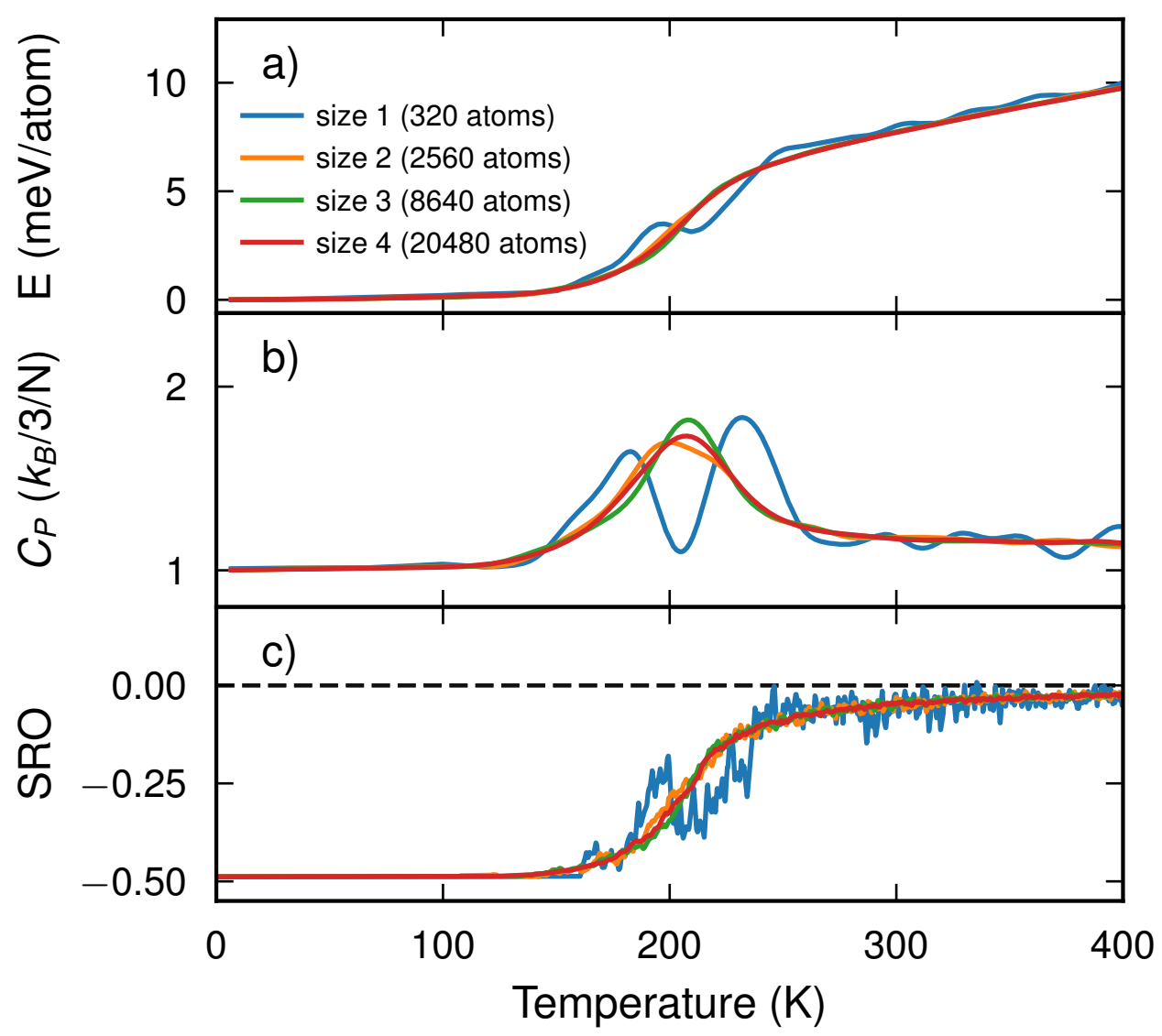

Figure S4: System-size dependence of the order–disorder transition for the perovskite (γ) phase at $x = 1/3$, using supercells containing 320, 2560, 8640, and 20 480 atoms. Shown are (a) the change in potential energy per atom, (b) the heat capacity, and (c) the chemical SRO parameter as functions of temperature. The transition behavior is consistent for the larger system sizes, demonstrating that the 8640-atom supercells used for the constant-temperature SGC simulations are sufficiently large to capture the order–disorder transition.

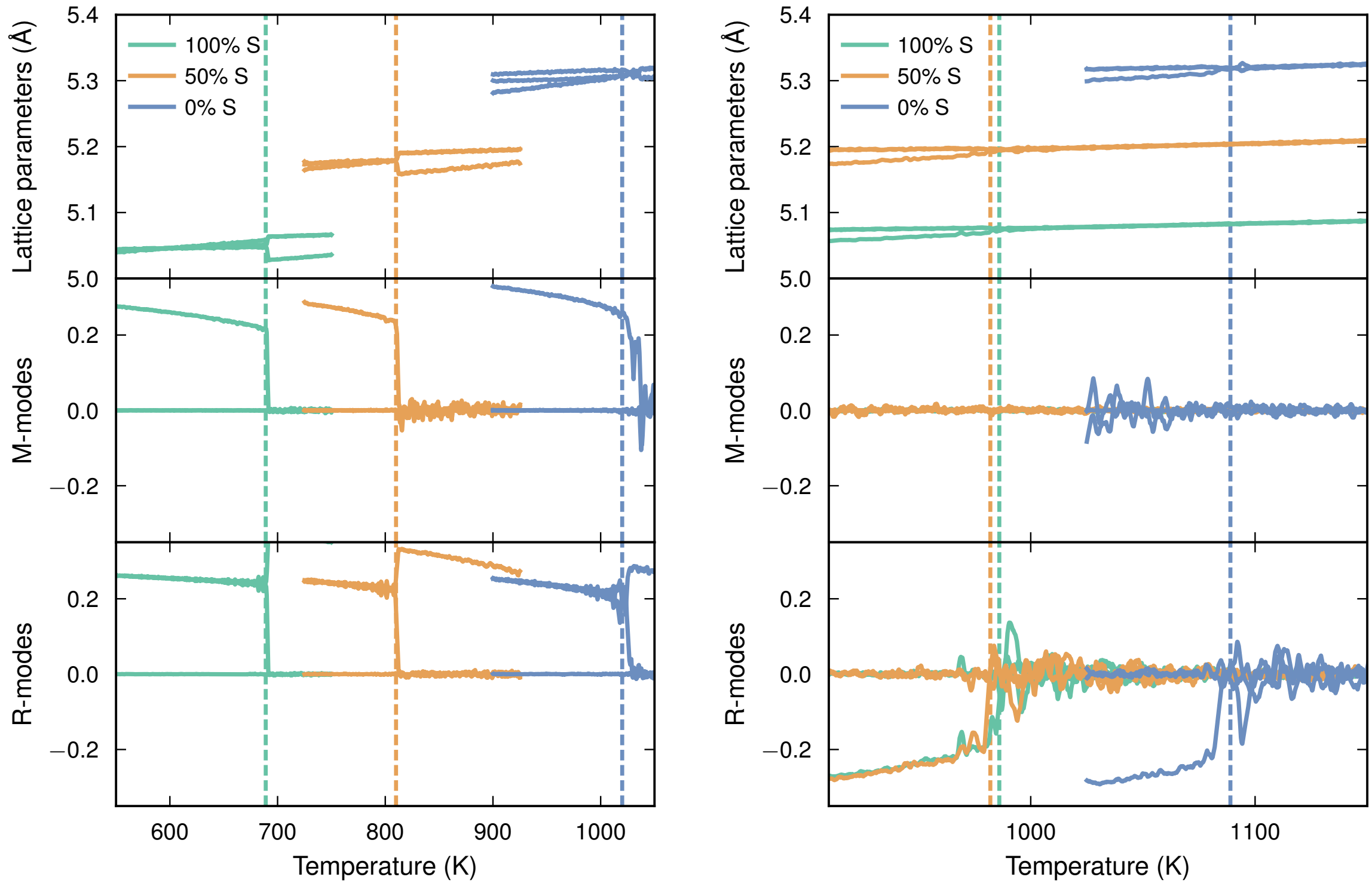

Figure S5: Phase transitions observed in MCMD simulations. The left panels show the orthorhombic-to-tetragonal transition, with the transition temperatures indicated by dashed vertical lines, while the right panels show the tetragonal-to-cubic transition.

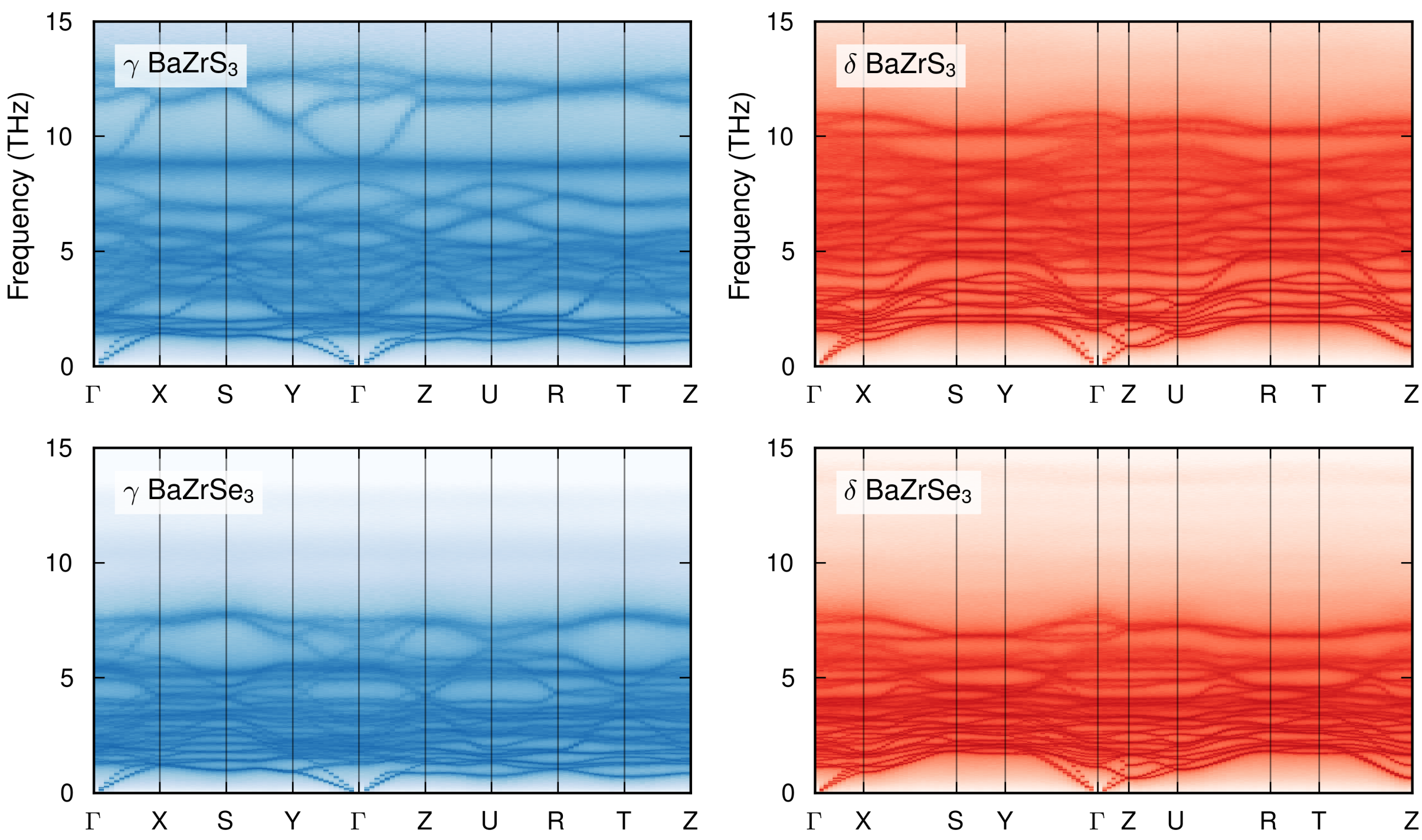

Figure S6: Phonon dispersions for δ and γ phases of $BaZrS_3$ and $BaZrSe_3$ at 300 K obtained from the spectral energy density (30) computed with DYNASOR (31, 32).

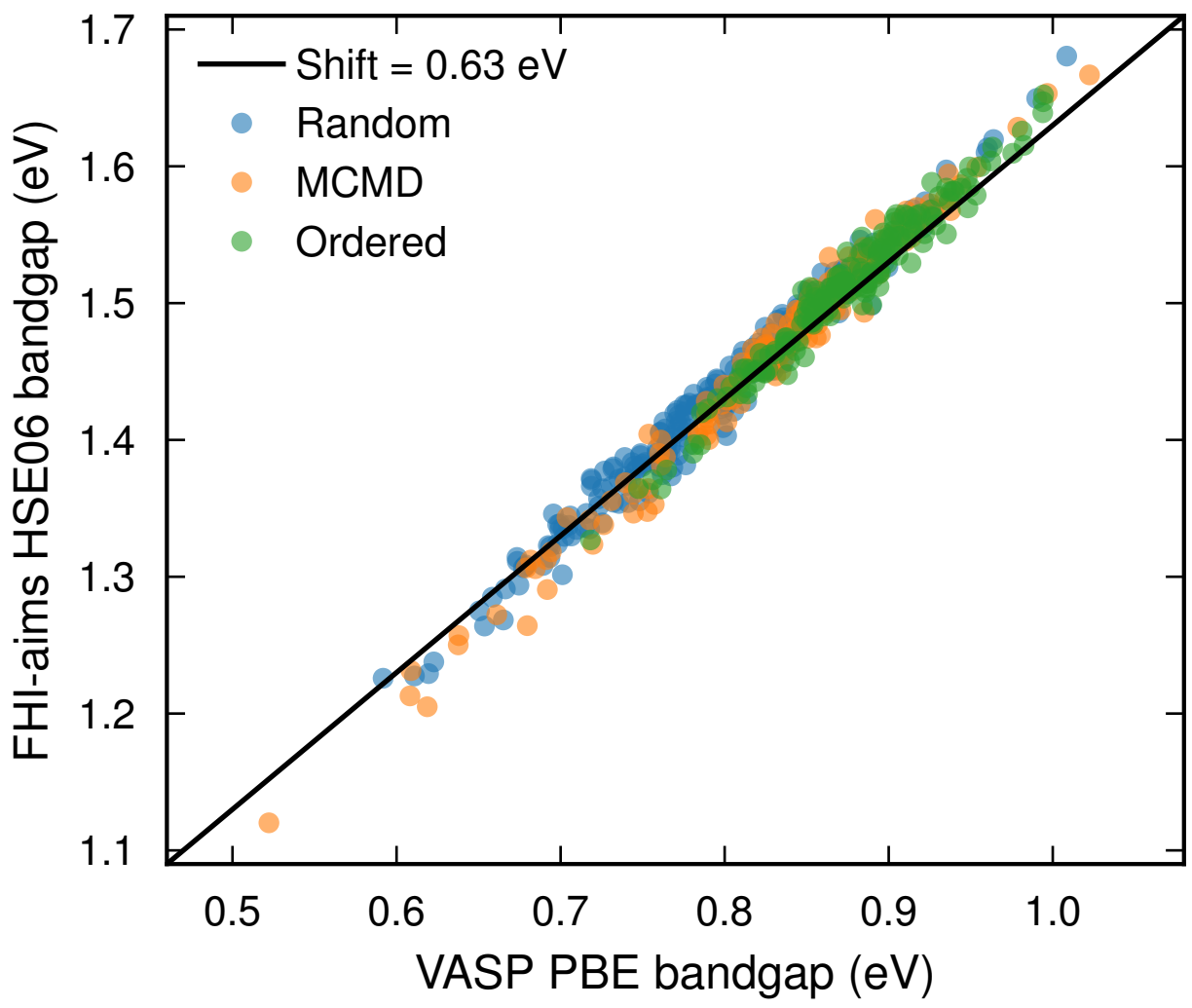

Figure S7: Comparison of PBE band gaps calculated using VASP and HSE06 band gaps calculated using FHI-AIMS for 40-atom snapshots. The snapshots were obtained at 100 K, 200 K and 300 K from simulations with random, equilibrated MCMD, and ordered anion configurations. The solid line corresponds to a rigid shift of 0.63 eV, demonstrating the approximately constant PBE-to-HSE06 band-gap correction used as the scissors correction for the calculated dielectric functions.

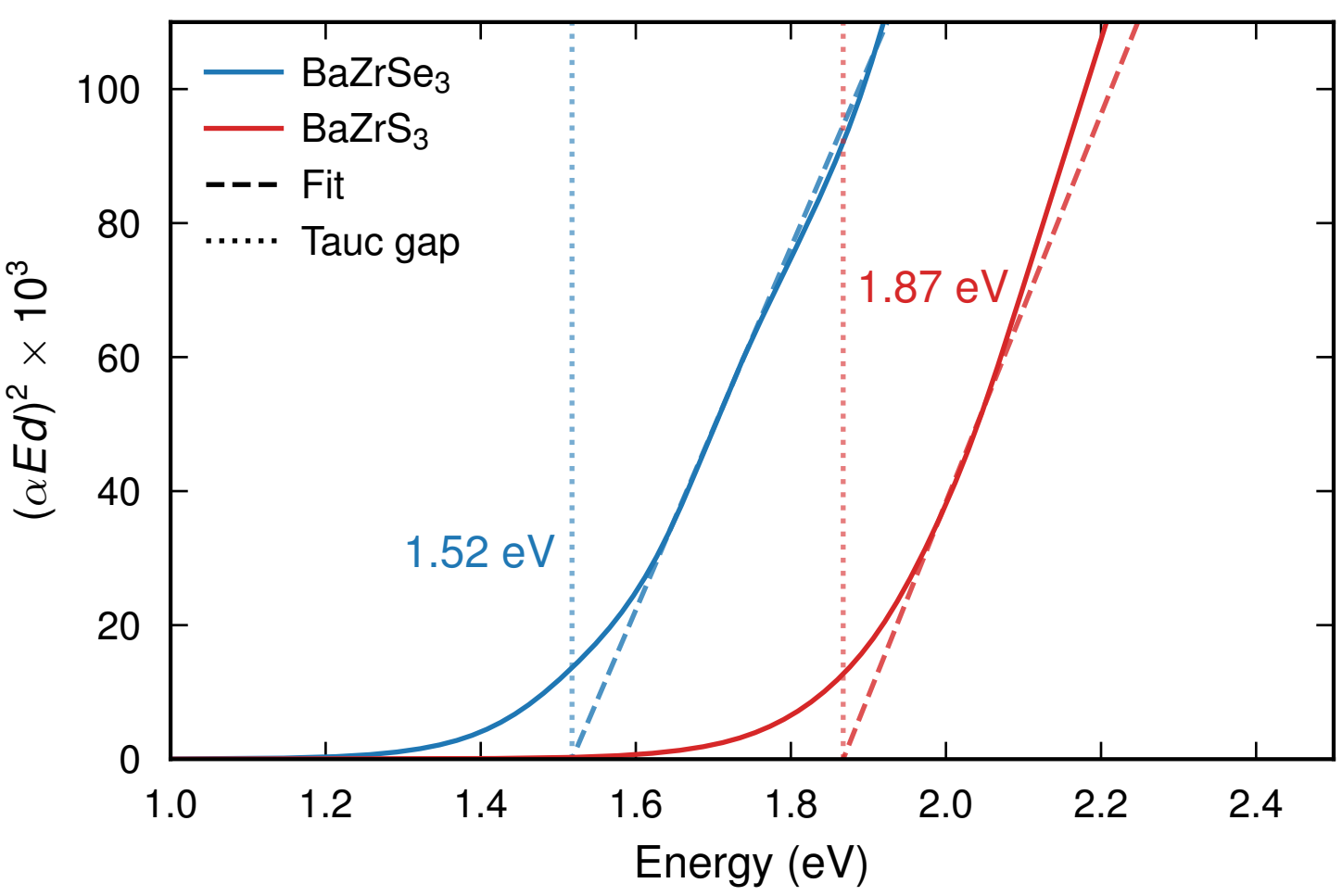

Figure S8: Fitting of the Tauc gap for pure $BaZrS_3$ and $BaZrSe_3$ at 300 K.

Supplementary Tables

Table S1: Comparison of the relaxed energy differences (0 K) for the relevant pure phases in units of meV/atom. The octahedral tilt patterns of the perovskite phases are described using Glazer notation.

		$BaZrSe_3$		$BaZrS_3$	
		NEP	DFT	NEP	DFT
Non-perovskite phases					
δ *Pnma*		0.0	0.0	0.0	0.0
$P6_3/mmc$		32.8	32.5	74.2	71.3
Perovskite phases					
γ *Pnma*	$a^-a^-c^+$	23.4	24.4	−1.6	−2.3
β *I4/mcm*	$a^0a^0c^-$	38.5	37.0	2.7	2.4
α $Pm\bar{3}m$	$a^0a^0a^0$	78.5	82.5	29.7	31.3

Table S2: Fundamental band gaps and Tauc gaps for $BaZrSe_3$ and $BaZrS_3$ in the different phases. The fundamental band gaps are calculated using HSE06 ($\alpha = 0.25$), while the Tauc gaps are extracted from PBE dielectric functions after application of the HSE06-derived rigid scissors correction. For the relaxed structures, fundamental band gaps are reported both without and with spin–orbit coupling, with the latter given in parentheses.

	Fundamental band gap (eV) relaxed	Fundamental band gap (eV) MD 300 K	Tauc gap (eV) MD 300 K
$BaZrSe_3$ perovskite	1.35 (1.18)	1.35	1.52
$BaZrS_3$ perovskite	1.81 (1.76)	1.66	1.87
$BaZrSe_3$ needle	0.98 (0.90)	1.04	1.95
$BaZrS_3$ needle	1.54 (1.53)	1.57	2.44

Supplementary References

[1] Z. Fan, Y. Wang, P. Ying, K. Song, J. Wang, Y. Wang, Z. Zeng, K. Xu, E. Lindgren, J. M. Rahm, A. J. Gabourie, J. Liu, H. Dong, J. Wu, Y. Chen, Z. Zhong, J. Sun, P. Erhart, Y. Su, and T. Ala-Nissila, *GPUMD: A package for constructing accurate machine-learned potentials and performing highly efficient atomistic simulations,* The Journal of Chemical Physics **157**, 114801 (2022). `doi:10.1063/5.0106617`.

[2] K. Xu, H. Bu, S. Pan, E. Lindgren, Y. Wu, Y. Wang, J. Liu, K. Song, B. Xu, Y. Li, T. Hainer, L. Svensson, J. Wiktor, R. Zhao, H. Huang, C. Qian, S. Zhang, Z. Zeng, B. Zhang, B. Tang, Y. Xiao, Z. Yan, J. Shi, Z. Liang, J. Wang, T. Liang, S. Cao, Y. Wang, P. Ying, N. Xu, C. Chen, Y. Zhang, Z. Chen, X. Wu, W. Jiang, E. Berger, Y. Li, S. Chen, A. J. Gabourie, H. Dong, S. Xiong, N. Wei, Y. Chen, J. Xu, F. Ding, Z. Sun, T. Ala-Nissila, A. Harju, J. Zheng, P. Guan, P. Erhart, J. Sun, W. Ouyang, Y. Su, and Z. Fan, *GPUMD 4.0: A high-performance molecular dynamics package for versatile materials simulations with machine-learned potentials,* Materials Genome Engineering Advances **3**, e70028 (2025). `doi:10.1002/mgea.70028`.

[3] A. H. Larsen, J. J. Mortensen, J. Blomqvist, I. E. Castelli, R. Christensen, M. Dułak, J. Friis, M. N. Groves, B. Hammer, C. Hargus, E. D. Hermes, P. C. Jennings, P. B. Jensen, J. Kermode, J. R. Kitchin, E. L. Kolsbjerg, J. Kubal, K. Kaasbjerg, S. Lysgaard, J. B. Maronsson, T. Maxson, T. Olsen, L. Pastewka, A. Peterson, C. Rostgaard, J. Schiøtz, O. Schütt, M. Strange, K. S. Thygesen, T. Vegge, L. Vilhelmsen, M. Walter, Z. Zeng, and K. W. Jacobsen, *The atomic simulation environment—a Python library for working with atoms,* Journal of Physics: Condensed Matter **29**, 273002 (2017). `doi:10.1088/1361-648X/aa680e`.

[4] F. Eriksson, E. Fransson, and P. Erhart, *The Hiphive Package for the Extraction of High-Order Force Constants by Machine Learning,* Advanced Theory and Simulations **2**, 1800184 (2019). `doi:10.1002/adts.201800184`.

[5] M. Ångqvist, W. A. Muñoz, J. M. Rahm, E. Fransson, C. Durniak, P. Rozyczko, T. H. Rod, and P. Erhart, *ICET – A Python Library for Constructing and Sampling Alloy Cluster Expansions,* Advanced Theory and Simulations **2**, 1900015 (2019). `doi:10.1002/adts.201900015`.

[6] G. L. W. Hart and R. W. Forcade, *Algorithm for generating derivative structures,* Physical Review B **77**, 224115 (2008). `doi:10.1103/PhysRevB.77.224115`.

[7] P. Kayastha, E. Fransson, P. Erhart, and L. Whalley, *Octahedral Tilt-Driven Phase Transitions in $BaZrS_3$ Chalcogenide Perovskite,* The Journal of Physical Chemistry Letters **16**, 2064 (2025). `doi:10.1021/acs.jpclett.4c03517`.

[8] E. Lindgren, M. Rahm, E. Fransson, F. Eriksson, N. Österbacka, Z. Fan, and P. Erhart, *calorine: A Python package for constructing and sampling neuroevolution potential models,* Journal of Open Source Software **9**, 6264 (2024). `doi:10.21105/joss.06264`.

[9] V. Blum, R. Gehrke, F. Hanke, P. Havu, V. Havu, X. Ren, K. Reuter, and M. Scheffler, *Ab initio molecular simulations with numeric atom-centered orbitals,* Computer Physics Communications **180**, 2175 (2009). `doi:10.1016/j.cpc.2009.06.022`.

[10] A. V. Krukau, O. A. Vydrov, A. F. Izmaylov, and G. E. Scuseria, *Influence of the exchange screening parameter on the performance of screened hybrid functionals,* The Journal of Chemical Physics **125**, 224106 (2006). `doi:10.1063/1.2404663`.

[11] G. Kresse and J. Furthmüller, *Efficiency of ab-initio total energy calculations for metals and semiconductors using a plane-wave basis set,* Computational Materials Science **6**, 15 (1996). Duplicate of KreFur96. `doi:10.1016/0927-0256(96)00008-0`.

[12] J. P. Perdew, K. Burke, and M. Ernzerhof, *Generalized gradient approximation made simple,* Physical Review Letters **77**, 3865 (1996). `doi:10.1103/PhysRevLett.77.3865`.

[13] P. E. Blöchl, *Projector augmented-wave method,* Physical Review B **50**, 17953 (1994). `doi:10.1103/PhysRevB.50.17953`.

[14] G. Kresse and D. Joubert, *From ultrasoft pseudopotentials to the projector augmented-wave method,* Physical Review B **59**, 1758 (1999). `doi:10.1103/PhysRevB.59.1758`.

[15] M. Moustafa, A. Ghafari, A. Paulheim, C. Janowitz, and R. Manzke, *Spin-orbit splitting in the valence bands of ZrS_xSe_{2-x}: Angle-resolved photoemission and density functional theory,* Journal of Electron Spectroscopy and Related Phenomena **189**, 35 (2013). 12th International Conference on Electronic Spectroscopy and Structure (ICESS-12). doi:10.1016/j.elspec.2012.12.010.

[16] R. Freitas, M. Asta, and M. de Koning, *Nonequilibrium Free-Energy Calculation of Solids Using LAMMPS,* Computational Materials Science **112**, 333 (2016). doi:10.1016/j.commatsci.2015.10.050.

[17] J. G. Kirkwood, *Statistical Mechanics of Fluid Mixtures,* The Journal of Chemical Physics **3**, 300 (1935). doi:10.1063/1.1749657.

[18] N. L. Allan, G. D. Barrera, M. Y. Lavrentiev, I. T. Todorov, and J. A. Purton, *Ab initio calculation of phase diagrams of ceramics and minerals,* Journal of Materials Chemistry **11**, 63 (2001). doi:10.1039/b002951n.

[19] M. Widom, W. P. Huhn, S. Maiti, and W. Steurer, *Hybrid Monte Carlo/Molecular Dynamics Simulation of a Refractory Metal High Entropy Alloy,* Metallurgical and Materials Transactions A **45**, 196 (2013). doi:10.1007/s11661-013-2000-8.

[20] J. M. Rahm and P. Erhart, *Understanding Chemical Ordering in Bimetallic Nanoparticles from Atomic-Scale Simulations: The Competition between Bulk, Surface, and Strain,* The Journal of Physical Chemistry C **122**, 28439 (2018). doi:10.1021/acs.jpcc.8b10874.

[21] E. Fransson, P. Rosander, F. Eriksson, J. M. Rahm, T. Tadano, and P. Erhart, *Limits of the phonon quasi-particle picture at the cubic-to-tetragonal phase transition in halide perovskites,* Communications Physics **6**, 173 (2023). doi:10.1038/s42005-023-01297-8.

[22] J. M. Cowley, *An Approximate Theory of Order in Alloys,* Physical Review **77**, 669 (1950). doi:10.1103/PhysRev.77.669.

[23] K. Ye, I. Sadeghi, M. Xu, J. Van Sambeek, T. Cai, J. Dong, R. Kothari, J. M. LeBeau, and R. Jaramillo, *A Processing Route to Chalcogenide Perovskites Alloys with Tunable Band Gap via Anion Exchange,* Advanced Functional Materials **34**, 2405135 (2024). doi:10.1002/adfm.202405135.

[24] I. Sadeghi, K. Ye, M. Xu, Y. Li, J. M. LeBeau, and R. Jaramillo, *Making $BaZrS_3$ Chalcogenide Perovskite Thin Films by Molecular Beam Epitaxy,* Advanced Functional Materials **31**, 2105563 (2021). doi:10.1002/adfm.202105563.

[25] L. Dieterle, B. Butz, and E. Müller, *Optimized Ar^+-ion milling procedure for TEM cross-section sample preparation,* Ultramicroscopy **111**, 1636 (2011). doi:10.1016/j.ultramic.2011.08.014.

[26] X. Sang and J. M. LeBeau, *Revolving scanning transmission electron microscopy: correcting sample drift distortion without prior knowledge,* Ultramicroscopy **138**, 28 (2014). doi:10.1016/j.ultramic.2013.12.004.

[27] J. M. Cowley and A. F. Moodie, *The scattering of electrons by atoms and crystals. I. A new theoretical approach,* Acta Crystallographica **10**, 609 (1957). doi:10.1107/S0365110X57002194.

[28] E. J. Kirkland, *Advanced computing in electron microscopy* (New York, NY: Springer, 2010). doi:10.1007/978-1-4419-6533-2.

[29] M. Xu, A. Kumar, and J. M. LeBeau, *Correlating local chemical and structural order using Geographic Information Systems-based spatial statistics,* Ultramicroscopy **243**, 113642 (2023). doi:10.1016/j.ultramic.2022.113642.

[30] J. A. Thomas, J. E. Turney, R. M. Iutzi, C. H. Amon, and A. J. H. McGaughey, *Predicting phonon dispersion relations and lifetimes from the spectral energy density,* Physical Review B **81**, 081411 (2010). doi:10.1103/PhysRevB.81.081411.

[31] E. Fransson, M. Slabanja, P. Erhart, and G. Wahnström, *dynasor—A Tool for Extracting Dynamical Structure Factors and Current Correlation Functions from Molecular Dynamics Simulations,* Advanced Theory and Simulations **4**, 2000240 (2021). doi:10.1002/adts.202000240.

[32] E. Berger, E. Fransson, F. Eriksson, E. Lindgren, G. Wahnström, T. H. Rod, and P. Erhart, *Dynasor 2: From simulation to experiment through correlation functions,* Computer Physics Communications **316**, 109759 (2025). doi:10.1016/j.cpc.2025.109759.
